# Mucosal Melanoma of the Oral Cavity: Prognostic Factors Influencing Patient Survival Outcomes

**DOI:** 10.3390/jcm14196863

**Published:** 2025-09-28

**Authors:** Jakub Tarnawski, Barbara Wojciechowska, Adam Polcyn, Łukasz Garbacewicz, Barbara Drogoszewska, Adam Michcik

**Affiliations:** 1 Department of Maxillofacial Surgery, University Clinical Centre in Gdańsk, Mariana, Smoluchowskiego 17, 80-214 Gdańsk, Poland; barbara.wojciechowska@gumed.edu.pl (B.W.); adampolcyn@gumed.edu.pl (A.P.); lgarbacewicz@gumed.edu.pl (Ł.G.); barbara.drogoszewska@gumed.edu.pl (B.D.); 2 Department of Maxillofacial Surgery, Faculty of Medicine, Medical University of Gdansk, 17 Mariana Smoluchowskiego Street, 80-214 Gdansk, Poland

**Keywords:** oral mucosal melanoma, melanocytic nevus, long-term follow-up, maxillo-facial surgery, oncological surgery

## Abstract

**Background/Objectives:** Oral mucosal melanoma is rare and associated with poor prognosis. This study aims to identify clinical and molecular factors influencing survival by synthesizing published cases together with one original case. **Methods:** A systematic review was conducted. Relevant clinical, histopathological, and follow-up parameters were compared across studies. **Results:** A total of 21 publications describing 42 patients were reviewed, and the data were aggregated with our patient, yielding 43 patients in total. Our case describing a 66-year-old male with 14 years of disease-free survival despite multiple excisions illustrated the potential for long-term survival despite poor prognosis. Favorable prognostic indicators listed in the literature included preserved expression of BAP1, p53, BCL2, and p16, the presence of tumor-infiltrating lymphocytes, smaller tumor size and thickness, and the absence of vascular invasion. Longer follow-up periods were most often observed in younger patients without lymphadenopathy, distant metastasis, or local recurrence. **Conclusions:** This review highlights prognostic features in oral mucosal melanoma but also underscores exceptions, demonstrating that survival cannot be fully explained by current clinical and molecular criteria.

## 1. Introduction

Primary mucosal melanoma is classified as a head and neck tumor by the National Comprehensive Cancer Network, with an incidence rate of 0.2 per million per year [1]. This type of lesions account for approximately 1.3% of all melanomas. The estimated five-year survival rate is 23% among patients aged 25–64 years, and the condition is frequently associated with local recurrence and distant metastasis [2]. While less than 2% of all melanomas are non-pigmented or amelanotic, the incidence of amelanotic melanomas is notably higher in the oral mucosa, reaching up to two-thirds of cases [3,4]. For an accurate diagnosis, an incisional biopsy is typically performed for histological evaluation. Because of its aggressiveness and high mortality rate, which result from rapid metastasis, early detection and intervention are critical. Surgical resection remains the primary treatment for malignant mucosal melanoma, although radiation therapy, chemotherapy, and immunotherapy may be used as adjuncts [5]. Given the limited number of reported cases, there is still a lack of comprehensive knowledge regarding the factors that contribute to prolonged post-surgical, cancer-free survival. This study’s scope was to find out which components are responsible for extension of the follow-up length among patients with oral mucosal melanoma.

## 2. Materials and Methods

A literature review was conducted to assess the current state of knowledge regarding survival in oral mucosal melanoma and its correlation with factors that are responsible for better prognosis. The search was performed using PubMed, MEDLINE, EMBASE, and the Cochrane Library. These databases were selected for their extensive coverage of biomedical, clinical, and multidisciplinary research. The following keywords were applied during the search process: oral mucosal melanoma, follow-up, prognostic factors, survival, and case study. Only articles describing clinical cases or case series were included. Filters were applied to select studies published between the years 1973 and 2025, encompassing both historical perspectives and the most up-to-date findings. Exclusion criteria were studies older than 1973, studies written in a language other than English, and studies that did not describe any of the factors listed in the tables. Studies were first screened based on a combination of their title and abstract. The full paper was then reviewed and included if no exclusion criteria were met. Selected cases were listed and compared in the two tables. The analyzed components were ones that are the most consistently available across the studies and relevant to survival prognosis, including the number of patients, type of treatment, follow-up length, time before the diagnosis, histopathological confirmation, age, sex, location and size of the tumor, recurrence, presence of lymphadenopathy, and metastasis. The risk of bias for the included studies was evaluated using the Newcastle–Ottawa Scale, a validated tool for assessing the methodological quality of observational studies. It consists of a maximum of 9 points across three key domains: Selection—4 points, Comparability—2 points, and Exposure/Outcome—3 points. Three reviewers performed the assessment. Studies scoring ≥ 7 were classified as low risk of bias, ones with 4–6 points as moderate risk, and those scoring < 4 as high risk of bias. The scale assessment revealed variability in methodological quality among the 21 included studies. Low risk of bias presented 6 studies, moderate risk of bias—10 studies, high risk of bias—5 studies. The distribution was balanced, 76% of the studies demonstrated a moderate or low risk of bias, primarily due to limitations in methodological details. Despite some methodological limitations, the data collected provides valuable insights into the oral mucosal melanoma theme. The supplementary table listing studies with its NOS score is available below (Table 1).

## 3. Results

The collected data are presented in two tables (Table 2).

Table 2 studies comparison by components including number of patients, type of treatment, follow-up length, time before the diagnosis, histopathological findings, age, sex, location and size of the tumor, recurrence, presence of lymphadenopathy, and metastasis. The dataset included 43 patients, 27 males and 16 females giving the 1.69 male to female ratio. One of the cases was our male patient. A 66-year-old male at first diagnosed in 2011 with a histopathologically confirmed oral mucosal melanoma. Over the following 14 years, he underwent seven surgical excisions of recurrent pigmented lesions in the oral cavity, some of which were oral mucosal melanomas. As of today, the patient remains cancer-free, and control diagnostic imaging shows no signs of local or distant recurrence. The patient remains under routine follow-up and represents a rare exception to the typical prognostic expectations reported in the literature. The uniqueness demonstrated by our patient included one of the longest follow-ups reported in this publication. Moreover, the patient did not develop distant metastases but presented nodal lymphadenopathy. Additionally, the patient was older and that is typically associated with a worse prognosis too. He also experienced multiple recurrences, and declined chemotherapy, all of which would normally suggest a poorer outcome. The studies included in abovementioned tables were also synthesized into a comprehensive tabular summary (Table 3).

**Figure 1:**
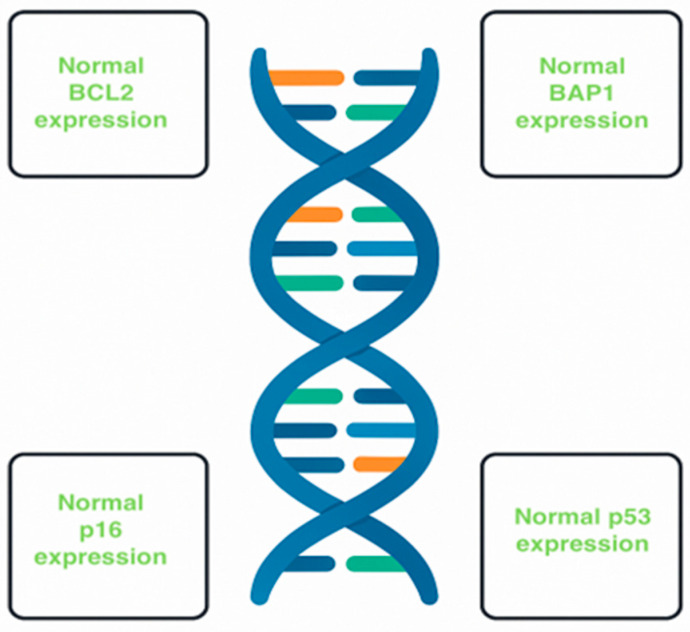
Molecular factors influencing the oral mucosal melanoma survival rate.

According to the literature, several positive prognostic factors for oral mucosal melanoma have been identified. Molecular ones included normal expression of BAP1, p53, BCL2, and p16 proteins (Figure 1). The presence of tumor-infiltrating lymphocytes plays an additionally important role, all of which have been linked to improved survival outcomes. Moreover, tumor size and especially smaller thickness, lack of vascular invasion, absence of lymph node involvement, lack of local recurrence, and the absence of distant metastases were also associated with better prognosis. Patient-related factors such as younger age, female sex, non-white race, and early diagnosis have also been correlated with reduced mortality and longer follow-up periods.

**Figure 2:**
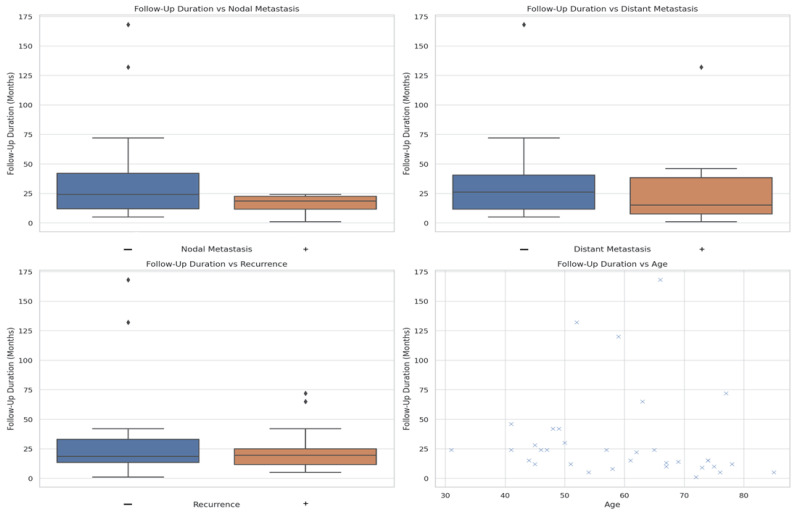
Visual analysis of collected data in three boxplots and one scatter plot graph. Comparison of recurrence and follow-up length; metastasis and follow-up length; lymphadenopathy and follow-up length; age and follow-up length. Absence of nodal metastasis, distant metastasis, and recurrence correlated with significantly longer survival. Age showed variable association although the literature often reports younger age as favorable, in this dataset some older patients demonstrated exceptionally long survival, underscoring individual variability.

The graphical results of the analysis of the data collected in the tables were presented in four charts (Figure 2). We observed a trend toward longer follow-up in patients without recurrence, with younger age, no lymphadenopathy, and no metastasis.

## 4. Discussion

The pathogenesis of mucosal melanoma is influenced by genetic, environmental, and immunological components. However, it does not have well-defined risk factors [26]. Several potential contributing elements include cigarette smoking, alcohol consumption, denture-related irritation, chronic microbial infections, chronic ulcerative stomatitis, mechanical stress from daily activities, and other factors that are harmful for the mucous membrane [27,28]. Unlike cutaneous melanoma, which is strongly linked to ultraviolet exposure, mucosal melanoma develops in areas that are not typically exposed to sunlight, making UV radiation an unlikely contributing factor. Melanocytes, the pigment-producing cells primarily responsible for UV protection and skin pigmentation, are also present in sun-shielded regions such as the oral cavity. While their exact function in these areas is not fully understood, evidence suggests they play a role in antimicrobial defense and immune response [29,30]. Although certain molecular alterations in genes such as c-KIT, BRAF, and NRAS have been identified in mucosal melanoma, their presence varies significantly among cases. Song et al. describe that DNA sequencing identified BAP1 missense mutations in 4 out of 12 patients with oral mucosal melanoma (OMM), while immunohistochemical analysis demonstrated a loss of nuclear BAP1 expression. This loss was associated with poorer overall survival and a higher incidence of distant metastases, establishing BAP1 as an independent prognostic marker in OMM [4,5,9]. Additionally, BCL-2 expression has been linked to more favorable prognostic outcomes in mucosal melanoma. In contrast, abnormal p53 protein expression and the absence of p16 protein expression have been correlated with a poorer prognosis [9,11]. These findings highlight the molecular heterogeneity of OMM, suggesting the presence of distinct molecular subtypes. Studies indicate no significant difference in the incidence of oral mucosal melanoma between men and women, though some reports suggest a slight predominance one over the other [31]. Additionally, men present a higher risk of mortality [32,33]. Despite sex difference, white race has been identified as an independent adverse prognostic factor for survival [34]. The literature has emphasized the importance of early diagnosis, as patients with localized or regional diseases demonstrate significantly better survival rates compared to those with distant metastases [35]. It also presents worse prognostic results for patients with pathological lymph nodes [32]. While this disease can affect adults of all ages, it is most commonly diagnosed in elderly individuals [36]. Although there is no definitive consensus regarding the correlation between age and prognosis, several studies suggest that older age is an unfavorable prognostic factor [34]. OMM is most often located in the region of the hard palate; the least common location is the uvula [37]. The first line of examination consists of the naked eye. In terms of more accurate screening, direct oral microscopy can be a useful tool; it derives from dermoscopy. This method can be used to see the mucosae at various sites such as the lip, cheek, floor of the mouth, ventral and lateral sides of the tongue, alveolar ridge, and soft palate. It allows for checking the subepithelial blood vessel patterns, mucosal surface, color tone, and transparency of the mucosa [38]. Because OMM can resemble benign pigmented lesions, inflammatory conditions, or other malignancies, it is crucial to perform a biopsy for any suspicious oral cavity lesion to confirm or rule out melanoma [39]. Histopathological analysis provides crucial prognostic insights, such as evidence of lymphatic and blood vessel invasion, both of which are associated with poorer outcomes. This information is vital for staging the disease using the TNM system. However, due to the aggressive nature of mucosal melanoma, cases involving epithelial or submucosal invasion without nodal metastases are classified as stage III, while deeper invasion into bone, nerves, skin, or lymph nodes is categorized as stage IV. Consequently, stages I and II do not apply to mucosal melanoma [2]. Oral mucosal melanoma can present in different patterns, including in situ, invasive, or a combination of both [8]. IHC does confirmation of the histopathological findings. In some cases, pigment may be entirely absent, making diagnosis reliant solely on immunohistochemistry. Tumor-associated stromal desmoplasia and chronic lymphocyte-dominated inflammatory infiltrates are commonly observed features, which help differentiate these tumors from melanocytic nevi. The presence of tumor-infiltrating lymphocytes serves as a positive prognostic indicator, while their absence has been linked to lymph node metastasis and a poorer prognosis. Lymphocytic infiltration is classified into three types: brisk, non-brisk, and absent. In the brisk category, tumor-infiltrating lymphocytes are observed throughout the vertical growth phase or covering the entire tumor base, most often in thin melanomas. Non-brisk infiltration refers to a localized presence of the cells within the tumor. In the absent category, lymphocytes are either completely missing or present without infiltrating the tumor tissue, this pattern is commonly associated with thicker melanomas and often linked to sentinel lymph node metastases. The degree of lymphocytic infiltration correlates with patient survival outcomes. In tumors with vertical growth, 10-year survival rates are approximately 55% for brisk, 45% for non-brisk, and 27% when these immunological cells are absent [5,31]. There are significant differences between melanoma cells and melanocytic nevi. OMM cells retain certain characteristics of melanocytic nevus, including a lack of dendritic extensions, a round to spindled morphology, and disrupted contact inhibition. However, these cancerous cells show marked pleomorphism, featuring large, irregular, hyperchromatic nuclei along with prominent nucleoli, and exhibit noticeable mitotic activity. Additionally, melanoma cells have the capacity to infiltrate the superficial mucosa in a Pagetoid pattern, invade the deeper submucosa, and spread through lymphatic and blood vessels to regional lymph nodes and distant organs [31,37]. Unlike CM, MM often lacks histologic landmarks such as the granular layer and subcutaneous tissue, rendering standard staging systems like Clark and Breslow inapplicable [6]. In 1997 WESTOP Banff Workshop classified OMM into three primary categories: in situ, invasive, and combined. The in situ variant, which accounts for 15% of cases, remains confined to the mucosal epithelium. The invasive type, representing 30% of cases, extends into the connective tissue beneath the mucosal layer. The combined pattern, observed in approximately 55% of cases, is associated with more advanced lesions [26]. Beyond the clinical stage, factors such as tumor thickness greater than 5 mm and vascular invasion detected via light microscopy, also are recognized as independent prognostic indicators in a study conducted by Patel et al. [36]. Once oral mucosal melanoma is diagnosed, whole-body computed tomography is typically recommended to differentiate between localized disease and occult metastases. Additionally, comprehensive tumor staging should include head and neck contrast-enhanced computed tomography, chest radiography, and liver function tests to assess elevated aspartate aminotransferase and alanine aminotransferase levels. Given that lung metastases are frequently observed in OMM, thoracic computed tomography is considered the most critical imaging modality for radiological surveillance in these patients [5]. The prevailing scientific consensus recommends complete surgical excision of the primary tumor, followed by postoperative adjuvant therapies. The most common is radiation therapy, which is nearly as widely used as chemotherapy; however, to date, for advanced cancers, chemotherapy remains the first line treatment. Chemotherapy was proposed to our patient but he rejected this therapeutic option. It is worth mentioning the importance of developing the field of targeted therapy. Until now there are only a few available targeted therapies for mucosal melanoma: BRAF, MEK, CDK4/6, and C-KIT inhibitors, with limited clinical use and efficacy. Immunotherapy includes immunomodulatory cytokines like interferon-alpha and interleukin-2, along with vaccines that target immune checkpoint molecules such as cytotoxic T-lymphocyte-associated protein 4 and programmed death-1 or its ligand, PD-L1. Research has demonstrated that it is generally more effective than traditional treatment methods such as chemotherapy or radiotherapy for post-surgical mucosal melanoma management [40,41,42,43,44,45]. The point of adjuvant therapies is to address microscopic or macroscopic residual disease or nodal involvement [46]. Even after complete surgical excision with negative margins, the recurrence rate of MM remains approximately 20% [5]. This underlines the importance of routine follow-up control. The recommended frequency of medical examinations for melanoma patients is not universally established, as recurrences may occur within five years of diagnosis, with some cases reported even more than a decade after surgical treatment [1]. The prevention and early detection of mucosal melanomas in the head and neck region involve yearly examinations for pigmented lesions within the oronasal cavity. Any pigmented area that does not correspond to an amalgam tattoo, racial pigmentation, or physiologic pigmentation caused by localized trauma, medications, or hormonal factors should be surgically removed and analyzed histologically. Early identification and removal of melanocytic lesions in these regions offer the best chance for a positive prognosis. The presence of amelanotic variants makes recognition particularly challenging, often resulting in diagnostic delays and unfavorable outcomes. Incorporating systematic screening into routine dental care for elderly patients, along with the use of diagnostic algorithms, may facilitate earlier detection and contribute to improved survival rates.

Raising awareness among healthcare providers and the general population about the importance of recognizing mucosal melanocytic lesions and melanomas in the head and neck may facilitate detection at an early, more treatable stage [37,46].

## 5. Conclusions

The study highlights the most common prognostic factors about oral mucosal melanoma that can be found in the publications. Moreover, analysis of collected data resulted in finding out the correlation between young age, absence of recurrence, lymphadenopathy, metastasis, and the length of follow-up. Several cases did not completely correspond with the results of our research and the literature statements, including the patient that we have described. Despite the absence of lymphadenopathy, distant metastasis, and an unclear molecular background, our patient did not align with other positive prognostic indicators identified in the literature. Nevertheless, he represented one of the longest follow-up periods recorded in this study, contradicting the typical prognostic expectations found in the literature. This underlines the need for further research in this field.

## Figures and Tables

**Table 1:** The supplementary table listing studies with its NOS score.

Author	Patients	Selection	Comparability	Outcome/Exposure	Total Score	Risk of Bias
Qamer et al., 2024 [6]	1	3	1	3	7	Low
Vos et al., 2022 [7]	1	3	1	3	7	Low
Bondi et al., 2021 [2]	1	2	1	2	5	Moderate
Jasper et al., 2022 [8]	1	2	1	2	5	Moderate
Correia-Neto et al., 2024 [9]	1	3	1	3	7	Low
Rosales et al., 1995 [10]	11	2	0	2	4	Moderate
Sen et al., 2021 [11]	1	1	0	2	3	High
Limongelli et al., 2020 [12]	1	2	1	2	5	Moderate
Becker et al., 2021 [13]	1	2	1	2	5	Moderate
Breik et al., 2016 [14]	1	3	1	2	6	Moderate
Carbone et al., 2018 [15]	1	3	1	3	7	Low
Cardoso et al., 2020 [16]	1	2	1	2	5	Moderate
Hajar-Serviansky et al., 2012 [17]	1	1	0	2	3	High
Horiuchi et al., 1994 [18]	1	1	0	2	3	High
Kemp et al., 2008 [19]	1	2	0	2	4	Moderate
Kuk et al., 2016 [20]	6	3	1	3	7	Low
Lourenço et al., 2009 [21]	1	2	1	2	5	Moderate
Luna-Ortiz et al., 2011 [22]	2	2	0	2	4	Moderate
Park et al., 2012 [23]	1	1	0	2	3	High
Sedassari et al., 2016 [24]	1	2	1	2	5	Moderate
Shastri et al., 2020 [25]	1	3	1	3	7	Low

**Table 2:** Information, including the number of patients, the type of treatment, follow-up time, time before diagnosis, histopathological findings, age, sex, tumor location and size, recurrence, presence of lymphadenopathy, and metastasis, was collected.

Author	Patients	Follow-Up	Treatment	Hist, Pat.	Age and Sex	Size	Time Before Diagnosis	Lymphadenopathy	Recurrence	Metastasis	Location
Reported case	1	14 y	partial maxillectomy with free radial flap reconstruction, adjuvant radiotherapy, and local radical resections	invasive and in situ	66 m	1.5 cm	2 mo	-	+ (multiple)	-	hard palate, maxillary alveolar process
ZahedAli Qamer et al., 2024 [6]	1	not reported	not reported	invasive and in situ	62 m	6 × 4.5 cm	1 y	+(1b + II + III)	not reported	+	hard palate
Teresa G. Vos. et al., 2022 [7]	1	5 mo	surgical	in situ	54 m	not reported	5 mo	-	+	-	hard palate and soft palate
Stefano Bondi et al., 2021 [2]	1	28 mo	surgical + radiotherapy	invasive + in situ pT3, pN0 stage III	45 m	not reported	5 y	+ (IIa)	-	-	hard palate and maxillary alveolar process
Polly Jasper et al., 2022 [8]	1	12 mo	surgical	in situ	45 f	2.2 × 2 × 1.4 cm	2 mo	-	-	-	left floor of mouth and lateral border of tongue
Ivan Jose CorreiaNeto et al., 2024 [9]	1	10 y	surgical + radiotherapy	not reported	59 f	not reported	not reported	not reported	not reported	not reported	left side of hard palate
J. Rosales et al., 1995 [10]	1	24 mo	surgical	not reported	65 f	not reported	not reported	not reported	+	not reported	hard palate
J. Rosales et al., 1995 [10]	1	12 mo	surgical	not reported	78 m	not reported	not reported	not reported	+	not reported	hard palate
J. Rosales et al., 1995 [10]	1	10 mo	surgical	not reported	75 f	not reported	not reported	not reported	-	not reported	hard palate
J. Rosales et al., 1995 [10]	1	15 mo	surgical	not reported	44 f	not reported	not reported	not reported	-	not reported	hard palate
J. Rosales et al., 1995 [10]	1	132 mo	surgical	not reported	52 m	not reported	not reported	-	+	+	hard palate
J. Rosales et al., 1995 [10]	1	9 mo	surgical	not reported	73 m	not reported	not reported	-	+	not reported	hard palate
J. Rosales et al., 1995 [10]	1	5 mo	surgical	not reported	85 f	2 × 1.5 cm	not reported	-	not reported	not reported	hard palate
J. Rosales et al., 1995 [10]	1	14 mo	surgical	not reported	69 m	5 cm	not reported	-	+	not reported	hard palate
J. Rosales et al., 1995 [10]	1	46 mo	surgical	not reported	41 m	3 cm	not reported	-	not reported	+	hard palate
J. Rosales et al., 1995 [10]	1	65 mo	surgical	not reported	63 m	0.5 cm	not reported	-	-	not reported	hard palate
J. Rosales et al., 1995 [10]	1	15 mo	surgical	not reported	61 m	5 cm	not reported	-	not reported	+	hard palate
J. Rosales et al., 1995 [10]	1	5 mo	surgical	not reported	76 f	2 cm	not reported	-	not reported	+	hard palate
J. Rosales et al., 1995 [10]	1	22 mo	surgical + radiotherapy	not reported	62 m	3 cm	not reported	-	+	not reported	hard palate
J. Rosales et al., 1995 [10]	1	24 mo	surgical	not reported	31 f	1 × 1 cm	not reported	-	-	not reported	hard palate
J. Rosales et al., 1995 [10]	1	24 mo	surgical	not reported	41 f	3 cm	not reported	-	-	not reported	hard palate
Suman Sen et al., 2021 [11]	1	not reported	not reported	not reported	59 m	3 × 3.5 cm	6 mo	-	not reported	-	hard palate
Luisa Limongelli et al., 2020 [12]	1	30 mo	surgical + chemotherapy	invasive	50 m	4 × 6 cm	3 mo	-	+	-	hard palate
Philipp Becker et al., 2021 [13]	1	2 y	surgical + radiotherapy	in situ	46 m	2 × 1.5 cm	not reported	-	+	-	hard palate, maxillary alveolar process
Omar Breik et al., 2016 [14]	1	not reported	surgical	not reported	57 m	1.6 cm	not reported	not reported	not reported	not reported	right buccal mucosa
M. Carbone et al., 2018 [15]	1	6 y	surgical	in situ	77 m	not reported	not reported	-	+	-	maxillary alveolar process
Diovana de Melo Cardoso et al., 2020 [16]	1	10 mo	surgical	in situ	67 f	3 × 1.5 cm	not reported	not reported	-	not reported	maxillary alveolar process
Tamar Hajar Serviansky et al., 2012 [17]	1	not reported	surgical	in situ	40 m	1.5 × 4 cm	not reported	not reported	not reported	not reported	lower lip
N Horiuchi et al., 1994 [18]	1	not reported	not reported	in situ	66 f	not reported	6 y	not reported	not reported	not reported	cheek
Spencer Kemp et al., 2008 [19]	1	38 mo	surgical	not reported	74 m	not reported	not reported	not reported	+	not reported	maxillary alveolar process, hard palate, cheek
Su KyungKuk et al., 2016 [20]	6	not reported	surgical	not reported	>58 (4), >58 (2). 3 m and 3 f	>0.5 cm (4), <0.5 cm (2)	not reported	not reported	not reported	not reported	maxilla 5, lip 1
Silvia Vanessa Lourenco et al., 2009 [21]	1	11 mo	surgical + chemotherapy	not reported	59 f	not reported	not reported	not reported	+	+	hard palate, maxillary alveolar process
Kuauhyama Luna-Ortiz et al., 2011 [22]	1	12 mo	surgical	not reported	56 m	not reported	not reported	not reported	-	+	lower alveolar ridge
Kuauhyama Luna-Ortiz et al., 2011 [22]	1	not reported	chemotherapy	not reported	37 f	not reported	not reported	not reported	-	+	lateral border of tongue
HyunSun Park et al., 2012 [23]	1	2 mo	surgical + chemotherapy	in situ, invasive	72 m	5.3 × 4.7 cm	10 y	+	+	not reported	upper lip
Bruno Tavares Sedassari et al., 2016 [24]	1	not reported	surgical	not reported	16 m	not reported	not reported	not reported	not reported	not reported	hard palate
Mayank Shastri et al., 2020 [25]	1	42 mo	surgical	not reported	49 m	3 × 2.5 cm	not reported	not reported	+	not reported	hard palate

**Table 3:** Aggregated analysis of collected data. This table summarizes key characteristics described in Table 2, including total number of patients, age range, location, lymphadenopathy, metastasis, recurrence, size range, follow-up range, time before diagnosis, and type of treatment. This table results in summed up purely numerical data that is associated with the 43 abovementioned patients. It helps to contextualize the heterogeneity of oral mucosal melanoma, demonstrating its variability.

Variable	Description
Total number of patients	43
Age	16–85 y
Sex	27 m (62.8%), 16 f (37.2%)
Location	Hard palate 28 (65.1%), maxillary alveolar process 12 (27.9%), soft palate 1 (2.3%), floor of mouth 1 (2.3%), tongue 2 (4.6%), buccal mucosa 3 (6.9%), lip 2 (4.6%), lower alveolar ridge 1 (2.3%)
Metastasis	8 (18.6%)
Recurrence	17 (39.5%)
Lymphadenopathy	3 (6.9%)
Time before diagnosis	2 mo- 10 y
Hist. pat.	Invasive 5 (11.6%), in situ 11 (25.6%)
Treatment	Surgical 25 (58.1%), surgical + radiotherapy 7 (16.3%), surgical + chemotherapy 3 (6.9%), chemotherapy 1 (2.3%)
Follow-up	2 mo- 14 y

## Data Availability

Data supporting reported results can be found in the tables from Section 2. All of the references are listed below.

## Abbreviations

The following abbreviations are used in this manuscript:

UV	Ultraviolet
CM	Cutaneous melanoma
OMM	Oral mucosal melanoma
IHC	Immunohistochemistry
